# Platelet-Rich Plasma from the Research to the Clinical Arena: A Journey Toward the Precision Regenerative Medicine

**DOI:** 10.3390/ijms27021058

**Published:** 2026-01-21

**Authors:** Elisabetta Mormone, Vittoria D’Esposito, Paola De Luca, Fulvio E. O. Ferrara, Francesca P. Bellotti, Pietro Formisano, Eugenio Caradonna

**Affiliations:** 1 Institute for Stem Cell Biology, Regenerative Medicine and Innovative Therapies (ISBReMIT), Fondazione IRCCS “Casa Sollievo della Sofferenza”, 71013 San Giovanni Rotondo, Italy; francesca_bellotti.577894@unifg.it; 2 Institute of Endotypes in Oncology, Metabolism and Immunology “G. Salvatore”—National Research Council (IEOMI-CNR), Via Pansini 5, 80131 Naples, Italy; vittoria.desposito@unina.it; 3 Orthopaedic Biotechnology Lab, IRCCS Ospedale Galeazzi-Sant’Ambrogio, Via Cristina Belgioioso 173, 20157 Milan, Italy; deluca.paola@grupposandonato.it; 4 Laboratory Medicine Department, CDI-Centro Diagnostico Italiano, Via Saint Bon 20, 20147 Milan, Italy; fulvio.ferrara@cdi.it (F.E.O.F.); eugenio.caradonna@cdi.it (E.C.); 5 Department of Translational Medicine, University of Naples “Federico II”, Via Pansini 5, 80131 Naples, Italy; pietro.formisano@unina.it; 6 Centro di Nanofotonica e Optoelettronica per la Salute dell’Uomo (CNOS), 82100 Benevento, Italy

**Keywords:** aging, anticoagulant, blood cells, centrifugation, cytokines, diet, growth factors, laboratory medicine, pre-analytical phase

## Abstract

Platelet-rich plasma (PRP) is a cornerstone of regenerative medicine, offering therapeutic potential across numerous clinical disciplines. Its efficacy relies on concentrated platelets and plasma components that release growth factors, cytokines, and extracellular vesicles to orchestrate tissue repair, immunomodulation, and angiogenesis. Recent findings have uncovered novel mechanisms, such as mitochondrial transfer from platelets to target cells and the delivery of bioactive microRNAs that regulate inflammation and metabolic reprogramming. However, despite its potential, PRP therapy is often limited by inconsistent results. In this review, we examine how patient-specific factors—including age, comorbidities, and lifestyle—and technical variables in preparation and storage, influence the biological quality of the final product. Therefore, standardizing protocols and accounting for individual biological variability are essential for achieving reproducible outcomes. In conclusion, PRP is a complex therapeutic agent whose success depends on both intrinsic bioactive content and extrinsic processing factors. Integrating these molecular insights with personalized patient assessment is crucial to optimizing PRP treatment procedures. Future research should focus on refining standardization to fully establish PRP as a precision medicine tool in regenerative therapy.

## 1. Introduction

Platelet-rich plasma (PRP) is considered one of the main pillars of regenerative medicine both in terms of its historical development and its widespread application across various clinical fields, including musculoskeletal conditions, gynecology, urology, plastic surgery, cardiac surgery, ophthalmology, and dermatology [1,2]. PRP is a biological product containing numerous bioactive molecules capable of regenerating cells, tissues, and organs [3]. The term PRP was introduced by hematologists in the 1970s to describe plasma with a platelet count higher than that of peripheral blood. It was initially used as a transfusion product to treat patients with thrombocytopenia. Later in the 1980s, researchers demonstrated clinical applications of PRP beyond hematologic conditions [4]. Nowadays, more than 1000 clinical trials are registered on ClinicalTrials.gov, over 40 PRP-based products are commercially available, and the number of therapeutic applications is constantly increasing [5]. However, despite its long-standing and very frequent use, there are still many concerns mainly related to the number of platelets, the presence of leukocytes and/or erythrocytes, the consistency of PRP (liquid or gel), the type of anticoagulant, the force and duration of the centrifugation [5,6]. These variables generate different PRP products and highlight the lack of standardization.

Although it has been shown that the regenerative effect of PRP is largely due to its platelet molecular content, some studies show that the plasmatic content—or extraplatelet molecules—including α2-macroglobulin and several growth factors, may play a fundamental role in various biological processes [7].

**Figure 1:**
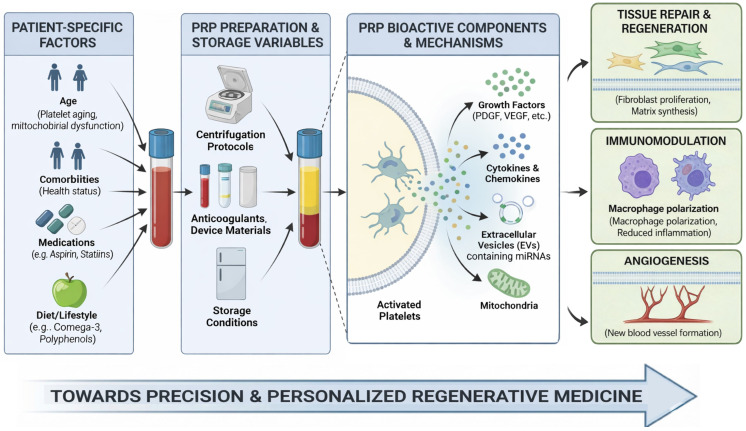
Flow chart summarizing the factors that may affect PRP composition and quality, from the patient to the preparation and storage.

Both plasma molecules and platelets are strongly influenced also by patient-dependent factors that have been very poorly investigated so far. Thus, in this review, we provide a brief overview of the novel molecular mechanisms of PRP action and focus on the patient factors, which can significantly affect the final PRP product. Elements such as age, overall health status, comorbidities, medication use, and even lifestyle choices (e.g., diet) can affect PRP composition and overall PRP quality. Finally, we address how methods of PRP preparation and storage (e.g., centrifugation, anticoagulants, materials) may impact platelet biology and the final PRP product (Figure 1). Understanding these variables is essential for optimizing treatment outcomes and tailoring PRP therapy to individual patient needs.

## 2. Methodology

### 2.1. Literature Search Strategy

A comprehensive literature search was performed using the PubMed/MEDLINE database to identify peer-reviewed articles investigating the biological properties, preparation techniques, and clinical applications of PRP. The search strategy was designed to encompass a broad range of topics, from basic cellular mechanisms to advanced clinical outcomes. The following Medical Subject Headings (MeSH) terms and keywords were used in various combinations: “Platelet-Rich Plasma”, “PRP”, “Growth Factors”, “Regenerative Medicine”, “Platelet Mitochondria”, “Extracellular Vesicles”, “Wound Healing”, “Aging”, “Diet” and “Pharmacological interaction”.

### 2.2. Data Extraction and Synthesis

The included sources span from foundational studies (dating back to the 1960s to provide historical context on platelet products) to the most recent evidence available up to 2025. Priority was given to recent systematic reviews, meta-analyses, and high-quality experimental studies that provide multi-omics or mechanistic perspective on how PRP promotes biological performance and tissue regeneration. We focused only on autologous PRP. All figures and tables were generated with the support of Artificial Intelligence tools.

## 3. Platelets and Regenerative Medicine

Platelets are small, discoid, anucleated cells that cannot duplicate, containing four types of granules (α, δ, λ, T) that house more than 1100 different proteins with numerous post-translational modifications, resulting in over 1500 protein-based bioactive factors [8,9]. These factors include immune system messengers, growth factors, enzymes and their inhibitors, and other factors which can participate in tissue repair and wound healing. α-Granules are the most abundant platelet granules with around 50–80 α-granules per thrombocyte. They constitute approximately 10% of platelet volume [10]. These granules contain membrane bound proteins, as well as more than 300 soluble proteins, which are released into the extracellular space [10]. These bioactive molecules are very heterogeneous and include proteins involved in clotting, inflammation, cell growth, cell adhesion and host defense [11]. Despite this rich bioactive reservoir, the final concentration of these factors is subject to significant inter-individual variability, primarily driven by the patient’s baseline blood count and metabolic status [12].

Moreover, different platelet sizes may reflect functional differences [13]. To quantify large platelets, automated cell counters can calculate the platelet large cell ratio (P-LCR), defined as proportion of platelets larger than 12 fL. Instead, no standardized parameter to quantify small platelets exists. Proteome analysis revealed that 80 of the 894 quantified proteins differed in abundance between large (Mean Platelet Volume-MPV 12.1 fL) and small platelets (MPV 7.7 fL). Specifically, ADP-ribosylation factor 1/3, guanosine triphosphate-binding protein SAR1a, Voltage-dependent anion-selective channel protein 3 and guanylate cyclase soluble sub-unit α-3 were more abundant in large, whereas immunoglobulins, haptoglobin, hemopexin, α-1-antitrypsin, serotransferrin and vitronectin were more abundant in small platelets [13]. This may suggest that small platelets modulate local and systemic inflammation at a larger extent compared with large platelets.

**Figure 2:**
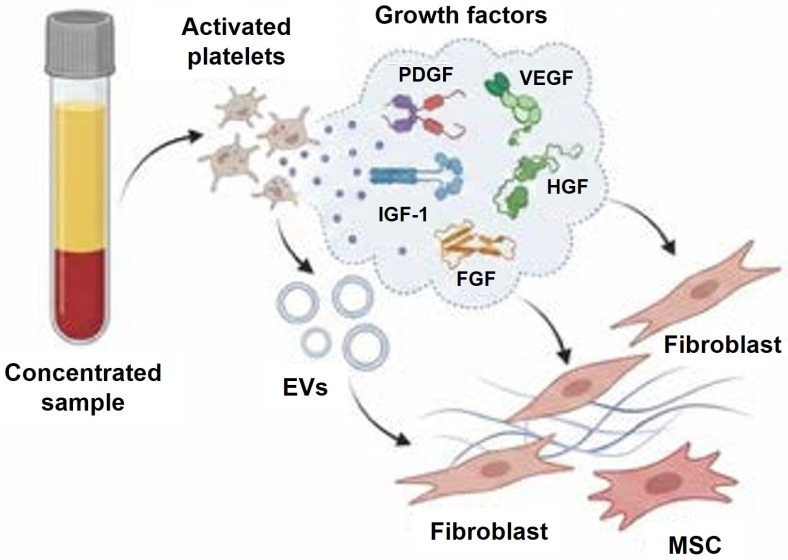
α-granules released by activated platelet secrete numerous factors which directly stimulate fibroblast proliferation, MSCs recruitment and ECM synthesis.

Following endothelial injury and disruption of the homeostasis, the platelets are activated. The α-granules are released in order to induce propagation signals and recruit additional platelets. α-Granules secrete several growth factors, including Platelet-Derived Growth Factor (PDGF) and Vascular Endothelial Growth Factor (VEGF) isoforms (namely, PDGF A, B, C, D and VEGF A, B, C), Insulin-like Growth Factor 1 (IGF-1), Hepatocyte Growth Factor (HGF), and Fibroblast Growth Factor (FGF). The released factors may directly stimulate fibroblast proliferation, mesenchymal stem cell (MSC) recruitment and synthesis of extracellular matrix (ECM), resulting in formation of fibrous connective tissue and scar deposition [14] (Figure 2). Circulating healthy platelets contain also a small number of mitochondria (about 4–8 per platelet) that play a variety of roles, from metabolism, activation, ATP production to regulation of cell processes and viability [15,16]. These mitochondria are metabolically active with a high rate of ATP turnover. The high levels of ATP are required for the normal functioning of ion channels that maintain the intracellular ionic balance, essential for preventing platelet activation [17]. It has been shown that larger platelets have higher abundances of mitochondrial proteins, suggesting increased metabolic capacity and higher potential to provide ATP [13]. In platelets, glycolysis provides about 60% of cellular ATP, while OXPHOS (mitochondrial ATP production) provides the remaining 30–40% [18]. Interestingly, platelets display metabolic flexibility that helps them meet energy demand. The ability to utilize glycolysis or fatty acid catabolism instead of OXPHOS allows platelets to adapt to different conditions, such as hypoxia or the presence of mitochondrial inhibitory agents [19]. Several studies have shown that platelet aggregation along with other metabolic activities are only fully interrupted when mitochondrial OXPHOS and glycolysis are inhibited simultaneously [20].

The metabolic pool of ATP and ADP is in the cytoplasm, whereas non-metabolic ATP and ADP are segregated into dense (δ) granules [21]. Platelet activation during standard plasma preparation led to the release of ~3.76 × 10^8^ full-length mtDNA copies per mL of plasma, which approximately correspond to about 40 to 1.25 × 10^8^ mitochondria per mL [22,23].

Moreover, in addition to cell-free mitochondria, platelet activation triggers cytoplasmic membrane budding and shedding of submicron vesicles called microparticles (MPs) [24]; considering the localization of mitochondria in the vicinity of the cytoplasmic membrane, it has been hypothesized that mitochondria may be packaged within MPs and form mitochondria-containing microparticles (mitoMPs) [25].

In general, MPs or microvesicles (MVs) are a type of extracellular vesicles (EVs) that vary in size between 100 and 1000 nm. On the other hand, exosomes are EVs with a smaller diameter ranging between 40 and 100 nm resulting from multivesicular body (MVB) packaging/endocytosis. Platelet EVs (pEVs) were historically identified as procoagulant particles released by activated platelets [26]. Later, it was suggested that the coagulant properties of pEVs are associated with microvesicles but not exosomes [27,28]. Moreover, pEVs carry molecules such as cytokines (e.g., IL [interleukin]-1β) [29], lipid mediators [30] and damage associated molecular patterns (DAMPs) [31], pointing to their role in the transfer of inflammatory and immune regulatory signals. pEVs cargo includes transcription factors, mRNA, and noncoding RNA [32]. Several studies have shown that pEV-derived miRNAs are incorporated into target cells and can signal with varying effects [33]. Therefore, although pEVs are produced by anucleated cells, they bear components capable of regulating the transcription, RNA stability, translation, and metabolism of their target cells. However, the study of EVs remains highly challenging due to the lack of validated methodologies for detection and quantification, as well as a lack of well-characterized reference standards.

## 4. PRP Mechanisms of Action

### 4.1. Canonical Mechanisms of Action

**Figure 3:**
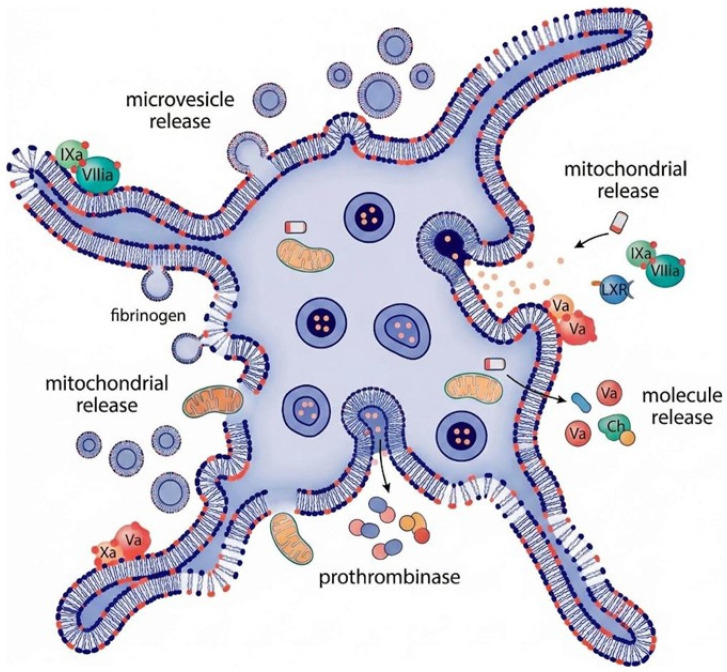
Schematic representation of platelet content.

Normal platelet count in healthy people ranges between 150,000 and 350,000/µL of blood. The administration of PRP creates a local 3–5-fold higher concentration of platelets which increases the blood supply and nutrient influx, necessary for the migration, proliferation, and differentiation of the cells involved in the healing process and neovascularization [2,34]. The most studied mechanism of action of PRP is based on the presence of numerous growth factors and cytokines in plasma and within platelet granules. Once PRP is administered, these molecules are released; hence, through specific receptors located on nearby and distant cells, they sustain all the cellular mechanisms related to the tissue regeneration. It has long been established that PRP contains and releases PDGF, IGF-1, VEGF, HGF, FGF, and various cytokines, including IL-6, IL-8, IL-10, and CCL5/RANTES [35,36] (Figure 3). Both in vitro and preclinical in vivo studies have extensively demonstrated that these molecules promote cell proliferation and migration, angiogenesis, collagen deposition, and modulation of inflammation [11]. For instance, PDGF enhances, macrophage activation, collagen synthesis, proliferation of bone cells, fibroblast chemotaxis and proliferative activity [11]. IGF-1 promotes cell growth, differentiation, recruitment in bone, blood vessel, skin and other tissues, stimulates collagen synthesis together with PDGF [11]. PRP-released IGF1 stimulates fibroblasts to produce IGF-1 in autocrine manner to activate ERK1/2 signaling and promote cell growth [37]. TGF-β enhances synthesis of type I collagen, promotes angiogenesis, stimulates chemotaxis of immune cells, inhibits osteoclast formation and bone resorption [11]. VEGF stimulates angiogenesis, migration and mitosis of endothelial cells, increases permeability of the vessels, stimulates chemotaxis of macrophages and neutrophils [11]. EGF stimulates cellular proliferation, differentiation of epithelial cells, promotes cytokine secretion by mesenchymal and epithelial cells [11]. FGF is a potent mitogen with multiple actions on multiple cell types; it promotes proliferation of mesenchymal cells (MSCs), chondrocytes and osteoblasts, stimulates the growth and differentiation of chondrocytes and osteoblasts [11]. Some of these factors as PDGF and TGF-β1 may decrease with age [38]. Clinical trials showed that this age-related decline can translate into a reduced clinical efficacy [39].

PRP also contains neurotransmitters such as serotonin, noradrenaline and BDNF, included in platelet α and dense granules, which may partially account for the analgesic effect of platelet concentrate [35] (Figure 3). Within the enzymes found in PRP there are superoxide dismutase (SOD), catalase (CAT), and glutathione peroxidase (GPx) [40] (Figure 3). In healthy tissue the presence of these antioxidant enzymes maintains physiological levels of reactive oxygen species (ROS) and minimizes the level of cellular stress; therefore, their presence in PRP products may contribute to the healing process keeping ROS levels low.

### 4.2. Novel Mechanisms of Action

More recently, novel mechanisms of action have been identified, further supporting the use of PRP in regenerative medicine. Preclinical in vivo studies have shown that PRP releases EVs enriched with various biomolecules, including microRNAs such as miR-26b-5p (Figure 3). This miRNA, targeting matrix metalloproteinase-8 (MMP-8), contributes to reducing neutrophil infiltration in wounds and promoting wound healing [41]. In vitro studies have also observed that activated platelets enhance wound closure by transferring mitochondria which modulate ROS levels in dermal fibroblasts [42]. In mesenchymal stem cells (MSCs), PRP-derived mitochondria induce metabolic remodelling by activating the fatty acid synthesis pathway, thereby stimulating MSCs to release pro-angiogenic factors into the vessel microenvironment [43]. Similar results were observed by Jin et al. [44] who found that platelets promote wound healing through releasing mitochondria taken up by vascular endothelial cells via dynamin dependent endocytosis. These data would suggest that PRP can provide respiratory-competent mitochondria to improve the efficacy of MSCs for the significant functional recovery of tissue injury and can reduce apoptosis caused by oxidative stress in vascular endothelial cells and in dermal fibroblasts [44]. In recent years, various research groups have investigated the effects of PRP on MSCs, both due to the potential benefits of their combined use in regenerative medicine and to understand the impact of platelet concentrate on local MSCs, which play a crucial role in tissue repair. In vitro studies, including transcriptomic and proteomic analyses, have clearly shown that PRP enhances MSCs biological performance by activating pathways related to proliferation, adhesion, survival, migration, and EVs release [45]. Moreover, PRP induces the expression of aquaporins on the MSC surface. These channel proteins, primarily involved in water transport, also play a role in cell motility [46]. PRP further regulates cellular differentiation mechanisms, preserving both adipogenic and osteogenic differentiation [47]. Notably, PRP has been shown to support MSCs osteogenic differentiation even in the presence of high glucose concentrations, a condition that typically hinders the differentiation process [48]. Finally, a recent systematic review going through 27 studies, including both in vitro and preclinical in vivo research, highlighted the PRP role in promoting a dynamic macrophage M1/M2 balance and facilitating a controlled M1-to-M2 transition over time [49]. Furthermore, platelets release the Platelet factor 4 (PF4), which may enhance the immune function of CD4^+^ central memory T cells, promoting mitochondrial biogenesis and T cell proliferation. This pathway enhances both Th1 (pro-inflammatory, via IL-17) and Treg (anti-inflammatory, via IL-10, TGF-β, IL-35) responses and also supports M2-like polarization of monocytes [50]. Thus, PRP serves as a reservoir of numerous bioactive factors that trigger multiple intracellular signaling pathways in target cells, ultimately driving tissue repair, immunoregulation, and pain modulation (Figure 3).

## 5. What Influences PRP Preparation?

During the formulation of PRP products, it is essential to study and consider the patient’s individual characteristics, number and quality of platelets, presence of white blood cells (WBC), methods of preparation, materials used, and storage conditions.

### 5.1. Interactions with Blood Cells

PRP, beyond being a platelet concentrate, often contains peripheral blood immune cells such as monocytes, neutrophils, and lymphocytes, depending on the preparation method. This cellular heterogeneity may contribute to its immunomodulatory properties, influencing the inflammatory environment and healing outcomes.

Among the key interactions between platelets and immune cells is the formation of platelet–monocyte aggregates (PMAs) [51]. These aggregates are initiated when activated platelets express P-selectin, which binds to the PSGL-1 receptor on monocytes. This molecular interaction triggers monocyte activation into either M1 (pro-inflammatory) or M2 (anti-inflammatory/regenerative) macrophage phenotypes and enhances the release of pro-inflammatory cytokines (TNF-α, IL-6, MCP-1), amplifying the immune response [49]. PMAs are now recognized as critical mediators at the intersection of hemostasis and inflammation, especially under thrombo-inflammatory conditions. Their levels are known to increase in diseases such as cardiovascular disorders, autoimmune diseases, and metabolic syndromes [52]. No evidence is currently available on the impact of PMAs in regenerative medicine applications. However, in the context of PRP, especially in leukocyte-rich preparations, the presence of these aggregates may influence the final composition and biological behavior of the concentrate, modifying both its regenerative and immunomodulatory effects. In addition, PRP can stimulate the formation of neutrophil extracellular traps (NETs). While NETs participate in host defence, they can also exacerbate oxidative stress and local inflammation. This has led to caution in the use of neutrophil-rich PRP (LR-PRP) in intra-articular or chronic conditions, with preference often given to leukocyte-poor PRP (LP-PRP) [53]. Upon activation, platelets release arachidonic acid, which is utilized by neutrophils to synthesize leukotrienes and prostaglandins, which are pro-inflammatory molecules. On the other hand, leukotrienes are absorbed from platelets to facilitate the production of lipoxins, which are potent anti-inflammatory agents [54,55,56].

These findings underscore that PRP should be characterized not only by platelet concentration but also by its leukocyte profile, which may significantly influence therapeutic outcomes.

### 5.2. Patient Characteristics

#### 5.2.1. Physiological Condition

The process of aging is a result of multiple interconnected biological processes that include DNA and protein damage, epigenetic changes, cellular senescence, stem cell exhaustion, systemic inflammation, and immune system decline [57]. Significant physiological changes occur within platelets, impacting their count and function. Aging is characterized by distinct populations of megakaryocyte progenitors and is associated with a peculiar proteomic profile of platelets. Platelets in older individuals display increased angiogenic protein expression, enhanced activity and are more prone to aggregation, leading to accelerated clot formation [58]. This increased activity is evidenced by heightened sensitivity to substances that induce platelet aggregation, such as ADP and epinephrine, and to serotonin [59]. Aging is also linked to increased oxidative stress, which affects platelet function [60]. ROS can enhance platelet activation, and oxidative stress can modify proteins and signalling pathways within platelets, resulting in their hyperactivity [61]. With age, the production of both thromboxane A2 (which promotes clotting) and prostacyclin (which inhibits clotting) increases, with the balance shifting towards a prothrombotic state. The composition of platelet cell membranes changes as well, including also an increase in cholesterol content. These changes can affect how platelets respond to signals and can lead to increased sensitivity to aggregation. Young platelets show higher mitochondrial content and membrane potential, as compared with older platelets, which, in turn, have reduced mitochondrial proteins, decreased cross-sectional area, and increased annexin V binding [62]. Thus, platelets transcriptome changes with age and influences how platelets function and interact with other cells, potentially contributing to platelet activity and inflammation [63]. However, the increased angiogenic protein expression of platelets from elderly could be one of the reasons of the efficacy of PRP in wound healing in these patients [64,65]. Understanding the interplay between aging and platelet characteristics is essential for optimizing PRP applications in older adults.

Furthermore, prevalent comorbidities in elderly individuals, such as cardiovascular diseases, obesity and diabetes, may further affect platelet activity [66,67]. Hyperglycaemia may induce ‘trained immunity’ in monocytes: AGEs and glucose prime cells epigenetically, leading to stronger inflammatory responses upon restimulation and promoting plaque formation [68]. Pharmacologic agents particularly used in the elderly, like statins, can reduce monocyte inflammatory cytokine production [69]. However, whether these conditions may affect regenerative potential of PRP is still under investigation to tailor PRP procedures on patients.

#### 5.2.2. Pharmacological Interactions

**Figure 4:**
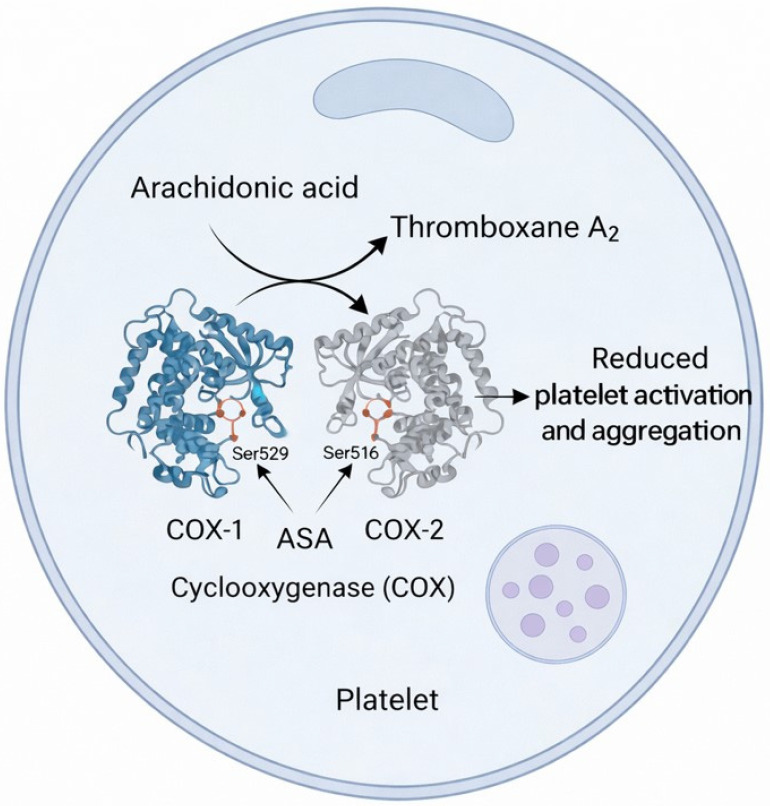
Schematic representation of ASA-mediated inhibition of PRP activation via COX-1 and COX-2 pathways.

Most patients scheduled for PRP treatment are concurrently undergoing pharmacologic therapy. The efficacy of regenerative therapy may be affected by the presence of certain medications. We focused on the mostly frequently encountered drugs in patients scheduled for PRP treatment. Notably, anti-inflammatory drugs, such as those targeting cyclooxygenase (COX) enzymes, can influence platelet function. COX enzymes exist in three distinct isoforms: COX-1, COX-2, and COX-3 [70]. COX-1 is generally considered the “constitutive” isoform, expressed regardless of inflammatory states and is particularly abundant in platelets, endothelial cells, gastric mucosa, and kidney tissue. Under physiological conditions, COX-2 is minimally expressed in mature platelets. This isoform is upregulated during the later stages of megakaryopoiesis. As a result, “young” or newly formed platelets entering the circulation may possess COX-2 derived from the cytoplasm of the progenitor megakaryocyte [71]. Acetylsalicylic acid (ASA) exhibits a distinctive mechanism of action among COX inhibitors, as it irreversibly inhibits both COX-1 and COX-2 through the acetylation of a specific serine residue (Ser529 in COX-1, Ser516 in COX-2) located within the hydrophobic channel of the enzyme’s active site (Figure 4). For these reasons, it is advisable to discontinue the use of ASA in patients scheduled for PRP treatment. If deemed necessary to continue ASA therapy, an alternative approach to activate PRP could be photobiomodulation or radiofrequency, which may induce platelet activation through mechanisms partially bypassing ASA’s inhibitory effects [72]. Statins therapy is often present in patients eligible for PRP applications. Multiple preclinical studies indicate that statins generally affect platelet function through different molecular pathways, besides conferring advantages in lipid and inflammation management. However, for patients who require minimal impact on platelet function, atorvastatin could be a preferable choice [73]. Metformin, a first-line drug for type 2 diabetes, appears to induce platelet dysfunction, suppressing intrinsic coagulation activity. This could lead to PRP treatment failure [74]. However, the regenerative potential of metformin should also be considered [75].

Regarding clinical guidelines, it is advisable to discontinue ASA 7–10 days prior to the preparation of PRP, while other nonsteroidal anti-inflammatory drugs (NSAIDs) should be suspended 48–72 h in advance. In cases where analgesia is required, paracetamol is the recommended alternative. Corticosteroids may inhibit the release of growth factors; therefore, their use should be halted one week before PRP treatment [76].

#### 5.2.3. Diet and Supplements

Dietary factors and nutritional supplementation may modulate platelet adhesion, aggregation, and recruitment function through different biological mechanisms, as evidenced by the extensive bibliography concerning dietary interventions in the treatment of thrombotic and thromboembolic disease [77] (Table 1). Many studies concern the inhibitory effects of diet or supplements on platelet aggregation, which may, in turn, diminish the therapeutic benefits of PRP treatment. In the supplementation context an example can be provided by a study in which magnesium in concentrations ranging from 1 mM to 10 mM exerts a significant, dose-dependent inhibitory effect on platelet aggregation and function [78]. Furthermore, since platelet activity is tightly regulated by nitric oxide (NO) produced by both platelets and endothelial cells [79,80], acute supplementation with nitrate salts has shown to reduce platelet reactivity. These effects were predominantly observed in male subjects, suggesting a potential sex-specific response that warrants further investigation [81]. Iron, a key component in hematopoiesis, is inversely associated with platelet counts [82]. While low iron stores may elevate platelet levels, current evidence does not robustly support a clinically meaningful impact of dietary or supplemental iron on PRP efficacy. Otherwise, Carica papaya leaf extract has shown promise in increasing platelet counts in preclinical models and in patients undergoing chemotherapy or with dengue fever [83,84]. Interestingly, also omega-3 fatty acid supplementation, specifically eicosapentaenoic acid (EPA) and docosahexaenoic acid (DHA), showed antiplatelet properties, as demonstrated in a systematic review of 52 studies [85]. Antioxidant vitamins influence platelet function by reducing oxidative stress. Among these Vitamin E (α-tocopherol) has dose-dependent effects: low doses have little impact, whereas higher doses significantly inhibit platelet aggregation [86,87]. In addition, vitamin C has similarly demonstrated a reduction in platelet activation at high doses (2 g), whereas lower doses (250 mg) did not produce statistically significant changes [87,88]. Policosanol, glucosamine, and polyphenols such as resveratrol, anthocyanins, and quercetin show antiplatelet effects, mainly at supplemental doses [89,90,91]. Human studies have shown that policosanol, anthocyanins, and quercetin are associated with decreased platelet aggregation and activation. Moreover, in vitro studies demonstrated that glucosamine and resveratrol inhibit platelet activity in a dose-dependent fashion in PRP samples [89,92]. In addition to food and supplements, also beverages can affect platelet aggregation [93]. Ginseng and its compounds, particularly ginsenosides Rg1, Rg2, and Rg3, exhibit strong antiplatelet effects.

Furthermore, the antiplatelet effects of the crude saponin fraction from ginseng on rat platelets were assessed. The findings demonstrated that the crude saponin fraction (ranging from 50 to 400 μg/mL) potently inhibited collagen-induced platelet aggregation by impeding calcium mobilization, ATP secretion, and fibrinogen binding to integrin αIIbβ3 [94]. Moreover, also green tea extract, which is rich in epigallocatechin-3-gallate (EGCG), also exerts the inhibition of platelet aggregation in PRP samples, as demonstrated in a recent study in which subjects took a placebo or GTE tablets (50 mg and 2 × 50 mg EGCG equivalent) daily for a period of two months [95]. The findings of this study suggest that in vitro treatment with green tea extract led to the inhibition of platelet aggregation in PRP of healthy subjects and of thalassemic patients. Thus, since numerous supplements, foods, and beverage may suppress platelet activity and potentially attenuate PRP efficacy, nutritional management of patients undergoing PRP treatments is crucial in optimizing clinical outcomes.

### 5.3. Methods of Preparation and Storage for Autologous PRP

#### 5.3.1. Centrifugation

**Figure 5:**
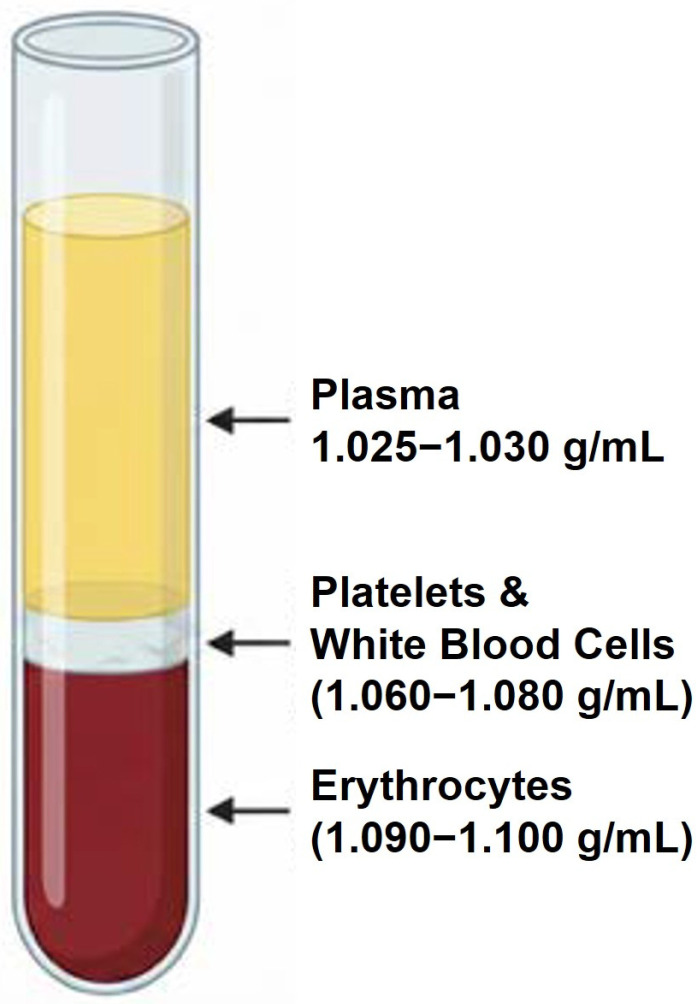
Gravity range for Plasma, White Blood Cells and Erythrocytes.

The preparation of PRP, according to Stokes’ law, depends on blood cells’ gravitational density variance and centrifugal force for separation. However, Stokes’ law applies to Newtonian fluid and spherical particles. The specific gravity of blood ranges from 1.048 to 1.066, while its density varies from 1.041 to 1.062 g/mL (or g/cm^3^). The specific gravity of whole blood is marginally higher in men compared to women and tends to increase following physical exercise and during nighttime. The circulating platelets are present in at least 14 populations with different density gravity ranging from 1.047 to 1.080 g/mL [96] (Figure 5). The difference in specific gravity reflects platelet age and indicates reduced granules, mitochondria, and proteins, characterized by diminished functionality and response to stimuli like calcium, thrombin, and collagen [62,97]. The mean platelet volume (MPV) is correlated with the mitochondrial and granule content of platelets [97]. MPV remains stable in healthy individuals over their lifetime with an inverse non-linear relationship with platelet count. Moreover, changes in platelet size were described for many disorders [98]. The mean protein concentration (MPC) (25–28 g/dL) represents the average protein content within platelets. In PRP, a low MPC is indicative of degranulated or activated platelets. High MPV and a low MPC suggest that the PRP is already activated. Lymphocytes are the second most abundant cell population present in PRP [99] with a specific gravity in the range 1.060–1.072 g/mL [35] (Figure 5). Based on their specific buoyant density, both T and B lymphocytes can be categorized into light and heavy types [100,101]. Monocytes constitute 2–8% of the WBC population with a gravity ranging from 1.062 to 1.068 g/mL [35]. Neutrophils are the most numerous WBCs representing 40–70% of the total population, their specific gravity is in the range 1.079–1.080 g/mL [102]. Recent evidence supports the presence of proinflammatory N1 with a low density (<1077 mg/mL) and heavier anti-inflammatory N2 (>1077 mg/mL). The different density gravity allocates the N2 at the border between the buffy coat and the red blood cells [103]. Characterization of leukocyte-rich PRP (LR-PRP) formulations have demonstrated that these preparations are predominantly lymphocyte-rich, with variable but notable concentration of other leukocyte subtypes, including neutrophils, monocytes, eosinophils, and basophils [99].

Eosinophils constitute approximately 0.5–1.5% of WBC and exhibit a higher density among leukocytes, measured at approximately 1.095 g/cm^3^ [104]. Basophils represent 0.2–1% of WBC with gravity ranges from 1.079 to 1.080 g/mL [105]. Erythrocytes typically have a diameter ranging from 6.7 to 7.7 micrometers (μm) and a specific gravity 1.090–1.100 g/cm^3^ [106]. Specific plasma gravity is reported in the range of 1.022 to 1.026 g/cm^3^. Thus, the buoyant density of platelets, lymphocytes, monocytes, and, to a certain extent, neutrophils fall within a similar range. As result, the complete isolation of platelets from WBCs—particularly monocytes—using bench-top centrifuges remains technically challenging.

Erythrocytes sedimentation ratio (ESR) represents a fundamental biophysical property of blood, specifically the propensity of erythrocytes to aggregate and sediment [107]. Factors influencing ESR, particularly plasma proteins mediating erythrocyte aggregation, affect PRP centrifugation separation dynamics. Therefore, the multiple factors determining ESR—protein levels, hematocrit, cell shape, age, sex, and medication—suggest significant variability in blood sedimentation dynamics, even in the absence of acute inflammation [108].

Therefore, it is plausible that a standardized centrifugation protocol, with fixed time and speed, may yield suboptimal results in terms of PRP yield or purity for some patients due to their specific basal hematologic and biophysical characteristics, partially reflected by the ESR value [108]. The “normal” range for HT is notably broad (e.g., 36–50% when considering both sexes). This physiological variability indicates that, even among “healthy” individuals, the initial conditions for PRP preparation will vary significantly in terms of plasma volume relative to red blood cells. This variability has direct implications for the volume of plasma that can be collected and the efficiency of platelet separation. For instance, if one patient has a HT of 36% and another has 50%, the former will possess a plasma volume of 64%, while the latter will have 50%. Consequently, for the same volume of blood drawn, the first patient provides a substantially larger volume of plasma. This difference immediately affects the potential volume of PRP obtainable and the concentration factor achievable with a standardized protocol. Standard centrifugation may yield significantly different PRP outcomes depending on the ESR and HT levels. This variability raises questions about the feasibility of applying a single “optimal” PRP protocol universally, even among patients with “normal” blood parameters. Consequently, this work does not encompass a description of the various centrifugation protocols.

#### 5.3.2. Anticoagulant

The anticoagulant mostly used in the preparation of PRP are sodium citrate and acid citrate dextrose (ACD-A, ACD-B) (Table 2). Sodium citrate (trisodium citrate dihydrate) functions as an anticoagulant by chelating calcium ions present in the blood. It is typically used at concentrations of 3.2% (0.109 M). In the 1930s, dextrose was incorporated into sodium citrate to supply energy, sustain cellular vitality, and prevent the hemolysis of red blood cells [109]. However, ACD-A, with a pH of 4.9, can cause pain at the site of injection. The presence of D-glucose induces proinflammatory effects [110]. The addition of 10% glucose creates a hyperglycemic environment for the cellular components of PRP. Regarding platelet recovery rates, sodium citrate appears to be superior to ACD-A [111]. Sodium citrate also enhances the release of exosomes from platelets compared to ACD-A [112]. Beyond conventional PRP, the evolution of platelet concentrates has led to the development of fibrin-rich matrices, such as Injectable Platelet-Rich Fibrin (i-PRF) and Concentrated Platelet-Rich Fibrin (c-PRF) [113]. Unlike traditional PRP, which requires exogenous anticoagulants, i-PRF and c-PRF are obtained through a low-speed centrifugation concept without additives.

#### 5.3.3. Materials Employed for PRP Preparation

**Figure 6:**
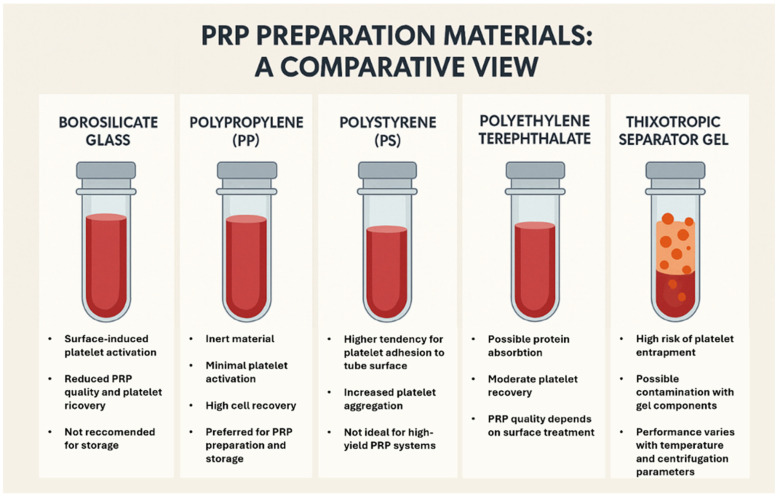
Tubes used for PRP preparation.

The surfaces of materials used in PRP preparation can significantly influence the composition and activation of PRP. The materials commonly used to produce tubes and devices for PRP preparation include glass, particularly borosilicate glass (Figure 6), and various medical-grade plastics such as polypropylene (PP), polystyrene (PS), and polyethylene terephthalate (PET) (Figure 6). Glass induces platelet activation via the intrinsic coagulation pathway, whereas plastics primarily achieve this through the adsorption of plasma proteins such as fibrinogen. In fact, platelets do not interact directly with the material but with the layer of adsorbed proteins that form on its surface. The composition and conformation of this protein film, particularly the balance between fibrinogen and albumin, influence platelet activation more than the material itself. When fibrinogen is denatured or changes conformation, it may expose binding sites for GP IIb/IIIa receptors, facilitating platelet adhesion, aggregation, and degranulation [114]. Because large platelets express 30 to 50% more GP compared with small ones, larger platelets could be more facilitated in the adhesion [98]. Some materials promote albumin adsorption, which can “passivate” the surface by reducing activation. This variability impacts PRP quality. PP is widely regarded as one of the most appropriate materials for the preparation and storage of platelets, as it appears to minimize the risk of premature activation or morphological alterations in platelets when compared to glass or PS. However, despite its favorable performance, standard PP is not entirely inert, as evidenced by the availability of “low protein-binding” and “low DNA-binding” versions, indicating a certain degree of protein adsorption even in standard materials. Platelet aggregation has been observed even in PP tubes, indicating a significant interaction. Regarding leukocytes, studies have detected their presence in Platelet Rich Fibrin (PRF) membranes prepared in PP, confirming that the material also facilitates the recovery of cellular components such as leukocytes and erythrocytes. PRF membranes derived from PS tubes also contain platelet aggregates. Thixotropic gels (Figure 6), which are inert polymeric substances, are extensively utilized in devices for the preparation of PRP due to their capacity to facilitate the separation of blood components during centrifugation [115]. The gel possesses a fixed specific density, which creates a distinct barrier separating the PRP located above from the erythrocytes and leukocytes below. Nevertheless, this fixed specific density is at odds with the density characteristics of the blood cells. The variation in temperature due to centrifugation speed and duration can alter the gel’s specific gravity, therefore platelet entrapment at the plasma–gel interface can significantly reduce platelet yield. Moreover, gels contain acrylates, nano-silica, and plasticizers, whose long-term biocompatibility if injected remains understudied. Furthermore, gel performance is influenced by environmental and patient variables (temperature, proteinemia, hematocrit), which affect plasma density.

#### 5.3.4. Storage

Storage methods, including temperature, storage duration, and activation methods, are key factors in the biological quality of PRP. Storage at 4 °C compared to the other temperatures analyzed (22 °C and −65 °C) appears to be the most effective in preserving platelet integrity and the availability of growth factors, representing a useful strategy in clinical settings where PRP cannot be used immediately [116]. Storage at −80 °C and −30 °C affects the protein structure and the mitochondrial membrane potential. Storage at −80 °C provides better protection of the mitochondrial structure and a lower level of ROS compared to −30 °C [117]. After 28 days, platelet activation increased significantly at both temperatures. However, no alterations in serotonin release capacity or macrophage activation were observed, suggesting that PRP cryopreserved at −80 °C for 14–28 days may maintain good functional properties in the clinical setting [117].

## 6. Summary and Limitations

### 6.1. Summary

PRP is a dynamic, autologous biological system rather than a standardized drug (Table 3). While its clinical potential in regenerative medicine is vast, its efficacy is inextricably linked to the interplay between patient-specific factors (age, pharmacology, lifestyle) and technical preparation variables (centrifugation, materials, anticoagulants). Beside the well-known functions of growth factors and cytokines, emerging evidence on mitochondrial transfer and pEV release provides a new mechanistic layer that explains how PRP orchestrates tissue repair and immunomodulation at a molecular level.

### 6.2. Limitations and Translational Gaps

Despite the promising results discussed, several limitations must be addressed to bridge the gap between bench and bedside in order to standardize the final product (Table 4):

- Methodological Bias: A significant portion of existing literature lacks rigorous standardization. The absence of detailed reporting on platelet concentration, leukocyte presence (LR-PRP vs. LP-PRP), and activation methods complicates the comparison between studies.
- Heterogeneity of PRP Products: The “PRP” acronym covers a wide range of products with large different biological profiles. This heterogeneity remains the primary obstacle to developing universal clinical guidelines.
- Patient-Specific Variability: As highlighted in this review, individual biological features (e.g., the use of ASA or metabolic status) are often overlooked in clinical trials, leading to inconsistent results and “non-responders.”
- Translational Gaps: Most “novel mechanisms” (e.g., mitochondrial transfer) have been predominantly observed in in vitro or animal models. High-quality, double-blind, randomized controlled trials (RCTs) focusing on these molecular pathways in humans are still lacking.

**Table 4:** Proposed Minimal Reporting Standards for PRP Characterization.

Parameter	Recommended Reporting	Impact on Clinical Outcome
Platelet Concentration	Absolute count (plt/µL) and Increase Factor over baseline.	Dose Dependence: Higher concentrations (e.g., >5x) are preferred for bone healing; lower concentrations might be better for hair restoration [118].
WBC Content	Presence/absence of Neutrophils (LR-PRP vs. LP-PRP).	Immunomodulation: Leukocytes may enhance the antimicrobial effect but can trigger excessive inflammation in joints (OA) [35].
EV Profile	Concentration and size of Extracellular Vesicle (EVs).	Paracrine Signaling: EVs mediate paracrine signaling and mitochondria transfer, influencing long-term metabolic tissue reprogramming [119].
Activation Method	Thrombin, Calcium Gluconate, Collagen or no activation.	Release Kinetics: Influences the kinetics of growth factor release (burst vs. sustained release) [120].
RBC Contamination	Percentage of residual RBCs.	Toxicity: High RBC content can lead to oxidative stress and synovial inflammation in intra-articular injections [121].

## Figures and Tables

**Table 1:** Effect of different supplements on platelet aggregation.

Category	Substance	Mechanism	Clinical Evidence	Refs
Minerals	Magnesium	Direct inhibition of platelet aggregation and function	In vitro studies	[53]
Minerals	Iron	Inversely associated with platelet counts	Review	[57]
Food additives	NO/Nitrate Salts	Enhanced NO-mediated antiplatelet signaling	Human studies (sex-specific response)	[54,55,56]
Plant Extracts	Carica papaya Leaf Extract	Increases platelet counts	Preclinical and clinical models	[58,59]
Omega-3 Fatty Acids	EPA & DHA	Antiplatelet signaling	Systematic review of 52 studies	[60]
Antioxidant Vitamins	Vitamin E	Dose-dependent inhibition	In vitro studies	[61,62]
Antioxidant Vitamins	Vitamin C	Reduces platelet activation	In vitro studies	[62,63]
Polyphenols	Policosanol	Antiplatelet effects	Human and in vitro studies	[64,65,66,67]
Polyphenols	Resveratrol	Dose-dependent antiplatelet activity	In vitro studies	[64,66,67]
Polyphenols	Anthocyanins	Antiplatelet effects	Human and in vitro studies	[64,65,66]
Polyphenols	Quercetin	Antiplatelet effects	Human and in vitro studies	[64,65,66]
Amino sugar	Glucosamine	Dose-dependent platelet inhibition	In vitro studies	[64,67]
Beverages	Ginseng & Ginsenosides (Rg1, Rg2, Rg3)	Inhibits platelet aggregation	In vitro studies	[68,69]
Beverages	Green Tea Extract (EGCG)	Inhibits platelet aggregation	Human studies	[70]

DHA: Docosahexaenoic Acid; EGCG: Epigallocatechin gallate; EPA: Eicosapentaenoic Acid; NO: Nitric Oxide; Rg1, Rg2, Rg3: Specific types of ginsenosides (active compounds in ginseng).

**Table 2:** Anticoagulant characteristics.

Anticoagulant	Anticoagulant Function	Additional Component	Platelet Recovery Rates	Exosome Release	Side Effects
Sodium Citrate	Chelates calcium ions present in the blood	None	Superior to ACD-A	Enhances the release of exosomes from platelets	None
ACD-A/ACD-B	Contains citrate (chelates calcium) plus dextrose (D-glucose)	Dextrose (D-glucose) supplies energy, sustains cellular vitality, prevents hemolysis	Inferior to sodium citrate	Inferior to sodium citrate	ACD-A can cause pain and inflammation at the injection site

ACD: acid citrate dextrose.

**Table 3:** Summary of PRP Bioactive Components, Mechanisms of Action, and Clinical Evidence Levels.

Category	Specific Component	Mechanism of Action	Level of Clinical Evidence	Clinical Relevance/Insights
Cellular Components	Platelets	Release of Growth Factors (PDGF, VEGF, IGF-1, HGF). Antioxidant enzymes (SOD, CAT, GPx); neurotransmitters (Serotonin, BDNF). Mitochondrial transfer to target cells.	High (Level I-II for Knee OA and Epicondylitis)	Fundamental for tissue repair, angiogenesis, and metabolic remodeling of MSCs.
	Leukocytes (WBCs)	Immunomodulation: M1 to M2 macrophage transition; formation of NETs (in LR-PRP).	Moderate (Context-dependent)	LP-PRP preferred for intra-articular use; LR-PRP for chronic tendinopathies.
Bioactive Molecules	Exosomes & pEVs	Delivery of microRNAs (e.g., miR-26b-5p) and mRNA to regulate target cell transcription.	Emerging (Strong pre-clinical data)	Key for long-distance signaling and sustained anti-inflammatory effects.
	Plasma Proteins	Antioxidant enzymes (SOD, CAT, GPx); α2-macrglobulin; neurotransmitters (Serotonin, BDNF), Growth Factors	Emerging	Reduction in oxidative stress, anti-inflammatory and potential analgesic effects post-injection.
Patient Factors	Physiological (Age/Diet)	Aging reduces mitochondrial potential; supplements (Omega-3, Vit E) inhibit aggregation.	High (Biological impact)	Nutritional management and age assessment are Crucial for reproducible outcomes.
Pharmacology	Drug Interactions (ASA, Metformin)	ASA irreversibly inhibits COX-1/COX-2; Metformin induces platelet dysfunction.	High (Clinical significance)	Medications must be discontinued or accounted for to prevent PRP treatment failure.

## Data Availability

No new data were created or analyzed in this study. Data sharing is not applicable to this article.

## Abbreviations

ACD-A/ACD-B	Acid Citrate Dextrose (solution A/B)
ADP	Adenosine Diphosphate
ASA	Acetylsalicylic Acid
ATP	Adenosine Triphosphate
BDNF	Brain-Derived Neurotrophic Factor
CAT	Catalase
COX	Cyclooxygenase (COX-1, COX-2, COX-3)
DAMPs	Damage-Associated Molecular Patterns
DHA	Docosahexaenoic Acid
ECM	Extracellular Matrix
EGCG	Epigallocatechin-3-gallate
EPA	Eicosapentaenoic Acid
ESR	Erythrocyte Sedimentation Ratio
EVs	Extracellular Vesicles
FGF	Fibroblast Growth Factor
GPIb-IX-V	Glycoprotein Ib-IX-V complex
GPx	Glutathione Peroxidase
HGF	Hepatocyte Growth Factor
IGF-1	Insulin-like Growth Factor 1
IL	Interleukin (es. IL-1β, IL-6, IL-8, IL-10)
LP-PRP	Leukocyte-Poor PRP
LR-PRP	Leukocyte-Rich PRP
MMP	Matrix Metalloproteinase (MMP-2, MMP-8, MMP-9)
MPC	Mean Platelet Component
MPV	Mean Platelet Volume
MSC	Mesenchymal Stem Cells
mtDNA	Mitochondrial DNA
NETs	Neutrophil Extracellular Traps
NO	Nitric Oxide
PDGF	Platelet-Derived Growth Factor (isoforms A, B, C, D)
PET	Polyethylene terephthalate
pEVs	Platelet-derived Extracellular Vesicles
PF4	Platelet Factor 4
PMAs	Platelet–Monocyte Aggregates
PP	Polypropylene
PRF	Platelet-Rich Fibrin
PRF-PP	Platelet-Rich Fibrin prepared in Polypropylene tubes
PRF-PS	Platelet-Rich Fibrin prepared in Polystyrene tubes
PRP	Platelet-Rich Plasma
PS	Polystyrene
ROS	Reactive Oxygen Species
SOD	Superoxide Dismutase
Th1	T helper type 1 cells
TNF-α	Tumor Necrosis Factor alpha
Treg	Regulatory T cells
VEGF	Vascular Endothelial Growth Factor (isoforme A, B, C)
WBC	White Blood Cells
